# CD4^+^ T Responses Other Than Th1 Type Are Preferentially Induced by Latency-Associated Antigens in the State of Latent *Mycobacterium tuberculosis* Infection

**DOI:** 10.3389/fimmu.2019.02807

**Published:** 2019-11-29

**Authors:** Yoshiro Yamashita, Toshiyuki Oe, Kenji Kawakami, Mayuko Osada-Oka, Yuriko Ozeki, Kazutaka Terahara, Ikkoh Yasuda, Tansy Edwards, Takeshi Tanaka, Yasuko Tsunetsugu-Yokota, Sohkichi Matsumoto, Koya Ariyoshi

**Affiliations:** ^1^Department of Clinical Medicine, Institute of Tropical Medicine, Nagasaki University, Nagasaki, Japan; ^2^Department of Respiratory Medicine, National Hospital Organization Higashi-Saga Hospital, Miyaki, Japan; ^3^Department of Respiratory Medicine, National Hospital Organization Nagasaki-Kawatana Medical Center, Kawatana, Japan; ^4^Food Hygiene and Environmental Health, Graduate School of Life and Environmental Science, Kyoto Prefectural University, Kyoto, Japan; ^5^Department of Bacteriology, Niigata University Graduate School of Medicine, Niigata, Japan; ^6^Department of Immunology, National Institute of Infectious Diseases, Tokyo, Japan; ^7^Tropical Epidemiology Group, London School of Hygiene and Tropical Medicine, London, United Kingdom; ^8^Infection Control and Education Center, Nagasaki University Hospital, Nagasaki, Japan; ^9^Department of Medical Technology, School of Health Science, Tokyo University of Technology, Tokyo, Japan; ^10^Laboratory of Tuberculosis, Institute of Tropical Disease, Universitas Airlangga, Surabaya, Indonesia; ^11^Department of Global Health, School of Tropical Medicine and Global Health, Nagasaki, Japan

**Keywords:** latent *M. tuberculosis* infection, CD4^+^ T cells, non-Th1, Acr, HBHA, MDP-1

## Abstract

*Mycobacterium tuberculosis* (*M. tuberculosis*) produces a diverse range of antigenic proteins in its dormant phase. The cytokine profiles of CD4^+^ T cell responses, especially subsets other than Th1 type (non-Th1 type), against these latency-associated *M. tuberculosis* antigens such as α-crystallin (Acr), heparin-binding hemagglutinin (HBHA), and mycobacterial DNA-binding protein 1 (MDP-1) remain elusive in relation to the clinical stage of *M. tuberculosis* infection. In the present study, peripheral blood mononuclear cells (PBMCs) collected from different stages of *M. tuberculosis*-infected cases and control PBMCs were stimulated with these antigens and ESAT-6/CFP-10. Cytokine profiles of CD4^+^ T cells were evaluated by intracellular cytokine staining using multicolor flow cytometry. Our results demonstrate that Th1 cytokine responses were predominant after TB onset independent of the type of antigen stimulation. On the contrary, non-Th1 cytokine responses were preferentially induced by latency-associated *M. tuberculosis* antigens, specifically IL-10 response against Acr in latent *M. tuberculosis* infection. From these results, we surmise a shift in the CD4^+^ T cell response from mixed non-Th1 to Th1 dominant type during TB progression.

## Introduction

One-quarter of the world's population harbors *Mycobacterium tuberculosis* (*M. tuberculosis*) (1), however, a majority of them are asymptomatic with the pathogen remaining dormant resulting in a latent *M. tuberculosis* infection (LTBI). Only 5–10% people with LTBI develop an active infection in their lifetime (2), which is responsible for two billion tuberculosis (TB) cases. The primary site of *M. tuberculosis* infection is almost exclusively alveolar macrophage in the lung thus the most common clinical form is pulmonary TB, contributing to efficient air-borne transmission. Furthermore, chronic nature of infection delays the patients from seeking adequate and timely health care (3). This time lag between the gradual onset of TB to the time of diagnosis and initiating treatment prolongs the critical period during with the patients are being infectious and spreading aerosolic *M. tuberculosis*.

Urgently needed is a diagnostic method that can detect TB before individuals with LTBI develop symptoms and become a source of infection. In the past two decades, IFN-γ release assays (IGRAs) have been developed and are widely utilized in clinical settings (4). This system quantifies the IFN-γ response to *M. tuberculosis*-specific antigens, which are expressed during active state, such as ESAT-6 and CFP-10, and detects the *M. tuberculosis* infection with a higher specificity than Mantoux skin test. However, IGRAs cannot distinguish active TB cases from LTBI (5), and current WHO policy discourages the use of IGRAs for the diagnosis of TB onset, especially in low- and middle-income countries (6). In reality, the predictive value for the development of TB from LTBI remains <10% (7).

The life cycle of *M. tuberculosis* is complex due to the dormant phase of the pathogen in the macrophages where it expresses a diverse range of latency-associated mycobacterial antigens: such as α-crystallin (Acr) (8), heparin-binding hemagglutinin (HBHA) (9), and mycobacterial DNA-binding protein 1 (MDP-1) (10). The active immune response against HBHA in LTBI has already been reported (11), however, to our knowledge, no previous reports described the various CD4^+^ T cell immune responses of multiple latency-associated antigens simultaneously.

CD4^+^ T cells are important components of TB granuloma and play a central role in restricting *M. tuberculosis* infection (12). Defective CD4^+^ T cell response in immune-deficient patients is reflected by the high burden of TB among HIV-infected population (13). The subsets of the CD4^+^ T cells are T-helper 1(Th1), Th2, Th17, and regulatory T cells (14–16) and these subsets have a distinct function, which either cooperate or interfere with each other. We believe that a comprehensive evaluation of wide range of T cell functions would be critical for better understanding the mechanisms involved in controlling *M. tuberculosis* infection, progression of latent infection to active TB, and the difference between latent infection and after-onset.

The objective of this study is to characterize the cytokine profile of the CD4^+^ T cell response to a range of *M. tuberculosis-*associated antigens, including multiple latency-associated antigens, in patients at the different clinical stages of TB, using multi-parameter flow cytometry. We observed that subsets other than Th1 type (non-Th1 type) CD4^+^ T cell responses were preferentially induced by latency-associated *M. tuberculosis* antigens in the state of LTBI.

## Results

### Study Participants

In total, 84 *M. tuberculosis*-infected cases and 19 healthy controls were recruited for the study. One infected case and one control were excluded due to inadequate blood sample, and one contact suspect case could not be confirmed by the QuantiFERON TB (QFT) test. Subsequently, 15 contact, 24 active, 24 on-treatment (median duration of treatment- 1 month) and 19 after-treatment cases (median duration of after treatment- 14 months) along with 18 healthy controls were evaluated. The characteristics of the participants are listed in Table 1.

**Table 1:** Characteristics of the participants.

**Characteristic**	**Contact (*n* = 15)**	**Active (*n* = 24)**	**On-treatment (*n* = 24)**	**After-treatment (*n* = 19)**	**Control (*n* = 18)**
**Age-year**					
Median	35.0	79.0	83.0	82.0	34.0
Range	22.0–57.0	32.0–95.0	60.0–99.0	34.0–93.0	26.0–80.0
**Male sex-no. (%)**	8 (53.3)	16 (66.7)	15 (62.5)	10 (52.6)	10 (55.6)
**TB type-no**.					
Pulmonary	–	21	21	15	–
Extra pulmonary	–	3	6	8	–
Both	–	2	3	4	–
**Clinical history-no. (%)**					
Diabetes	0 (0)	5 (21)	4 (17)	0 (0)	0 (0)
Malignancy	0 (0)	2 (8)	6 (25)	3 (16)	1 (6)
Collagen/allergy	1 (7)	2 (8)	3 (13)	1 (5)	1 (6)
Chronic resp disease	1 (7)	1 (4)	1 (4)	5 (26)	0 (0)
Other	2 (13)	12 (50)	15 (63)	12 (63)	1 (6)

The median age of all *M. tuberculosis*-infected cases was 77.5 years. Actively infected cases were older, and more likely associated with TB risk factors such as diabetes or malignancy, than the contact cases and controls. There was no significant difference in the percentage of male between groups.

### Th1 Cytokine Response of CD4^+^ T Cells to a Range of *M. tuberculosis*-Associated Antigens

**Figure 1:**
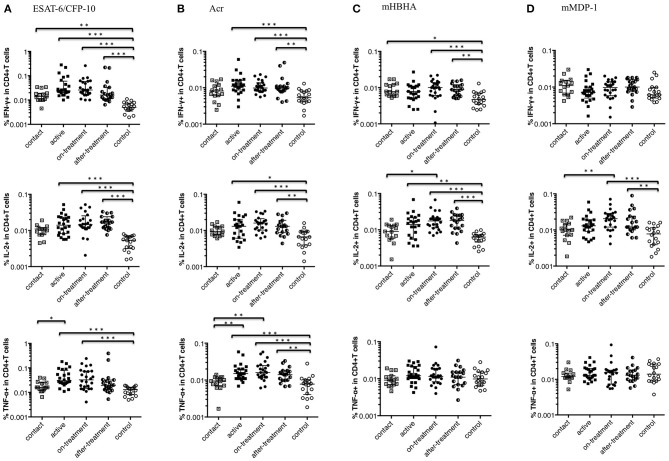
Th1 cytokine (IFN-γ, IL-2, and TNF-α) responses of CD4^+^ T cells to *M. tuberculosis*-associated antigens. Th1 cytokine responses of CD4^+^ T cells in *M. tuberculosis*-infected cases, grouped according to the disease stage as active TB cases (*n* = 24), on-treatment TB cases (*n* = 24), after-treatment TB cases (*n* = 19), and contact cases (*n* = 15). Responses of control cases (*n* = 18) are also shown. The differences between each set of samples were assessed using the Kruskal-Wallis test and *post-hoc* Dunn's Comparison test (*P < 0.05, **P < 0.01, ***P < 0.001). The long horizontal line represents the median and the vertical line represents the interquartile range. **(A)** Th1 cytokine responses to ESAT-6/CFP-10 (One data point is outside the limits in IFN-γ, and also one data point is outside the limits in IL-2). **(B)** Th1 cytokine responses to Acr (Three data points are outside the limits in IL-2). **(C)** Th1 cytokine responses to methylated (m) HBHA (Two data points are outside the limits in IL-2). **(D)** Th1 cytokine responses to mMDP-1.

Figure 1 shows Th1 cytokine (IFN-γ, IL-2, and TNF-α) responses of CD4^+^ T cells to a range of *M. tuberculosis*-associated antigens in active, on-treatment, after-treatment, and contact TB cases together with healthy controls. The level of Th1 cytokine responses was significantly higher among TB cases than controls. In more details, the highest IFN-γ and TNF-α responses against ESAT-6/CFP-10 or Acr were observed in after-onset TB cases (active, on-treatment, and after-treatment cases) (Figures 1A,B), whereas the IFN-γ response against methylated (m) HBHA was less apparent (Figure 1C). The level of Th1 cytokine responses in contact cases was generally lower than after-onset TB cases, and the difference was significant in IL-2 response against mHBHA and mMDP-1 (Figures 1C,D), and in TNF-α response against ESAT-6/CFP-10 and Acr (Figures 1A,B). It was only in IFN-γ response against ESAT-6/CFP-10 and mHBHA where the significantly higher level of cytokine responses was detected among contact cases than controls. These findings are comparable with results of previously published studies on IGRAs; indicating IFN-γ response alone is unable to detect TB clinical stage. IL-2 responses were remarkably strong among treated TB cases (on-treatment and after-treatment cases) (Figures 1A–D). Interestingly, a similar pattern of Th1 type T cell responses were observed irrespective of the type of mycobacterial antigen.

### Non-Th1 Cytokine Response of CD4^+^ T Cells to a Range of *M. tuberculosis*-Associated Antigens

**Figure 2:**
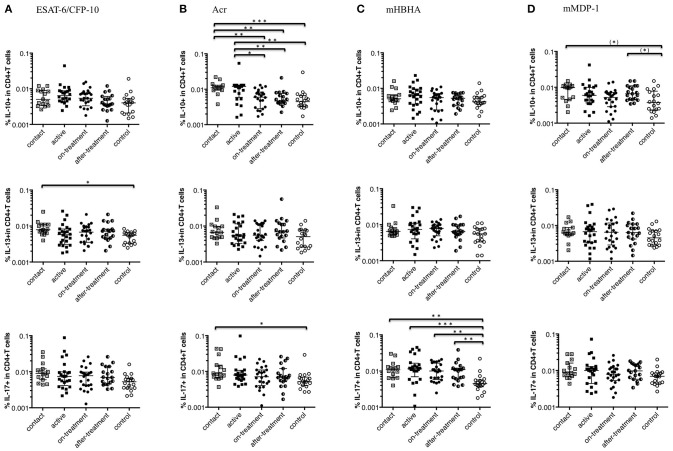
Non-Th1 cytokine (IL-10, IL-13, and IL-17) responses of CD4^+^ T cells to *M. tuberculosis*-associated antigens. Non-Th1 cytokine responses of CD4^+^ T cells in *M. tuberculosis*-infected cases, grouped according to the disease stage as active TB cases (*n* = 24), on-treatment TB cases (*n* = 24), after-treatment TB cases (*n* = 19), and contact cases (*n* = 15). Responses of control cases (*n* = 18) are also shown. The differences between each set of samples were assessed using the Kruskal-Wallis test and *post-hoc* Dunn's Comparison test (*P < 0.05, **P < 0.01, ***P < 0.001). (*) means the possible differences of preselected pairs (*P < 0.05). The long horizontal line represents the median and the vertical line represents the interquartile range. **(A)** Non-Th1 cytokine responses to ESAT-6/CFP-10 (Three data points are outside the limits in IL-10, and also three data points are outside the limits in IL-13). **(B)** Non-Th1 cytokine responses to Acr (Three data points are outside the limits in IL-10, and also three data points are outside the limits in IL-13). **(C)** Non-Th1 cytokine responses to methylated (m) HBHA. **(D)** Non-Th1 cytokine responses to mMDP-1 (One data point is outside the limits in IL-10, one data point is outside the limits in IL-13, and one data point is outside the limits in IL-17).

Figure 2 depicts non-Th1 cytokine (IL-10, IL-13, and IL-17) responses of CD4^+^ T cells to a range of *M. tuberculosis*-associated antigens in *M. tuberculosis*-infected cases and healthy controls. In contrast to the Th1 cytokine responses described above, the level of non-Th1 cytokine responses among after-onset TB cases (active, on-treatment, and after-treatment cases) was not significantly different (higher) than the controls except for IL-17 responses against mHBHA (Figure 2C). A significantly higher level of non-Th1 cytokine responses was detected among contact cases than controls; in IL-10 responses against Acr (Figure 2B), IL-13 responses against ESAT-6/CFP-10 (Figure 2A), IL-17 responses against Acr and mHBHA (Figures 2B,C). IL-10 responses against mMDP-1 were remarkably higher among the contact and after-treatment cases than controls (Figure 2D). Intriguingly the level of IL-10 responses against Acr was significantly higher among contact cases than on-treatment and after-treatment TB cases (Figure 2B). We used Linear regression analysis to adjust the association between factors such as age, diabetes, and malignancy (Table 1). Although malignancy was associated with a difference in IL-13 responses against ESAT-6/CFP-10, IL-13 responses were still higher among contacts than controls after correcting for malignancy. No evidence in the association of these factors with other significantly different non-Th1 cytokine responses was noted.

### Th1 Polyfunctional CD4^+^ T Cells in Response to *M. tuberculosis*-Associated Antigens

**Figure 3:**
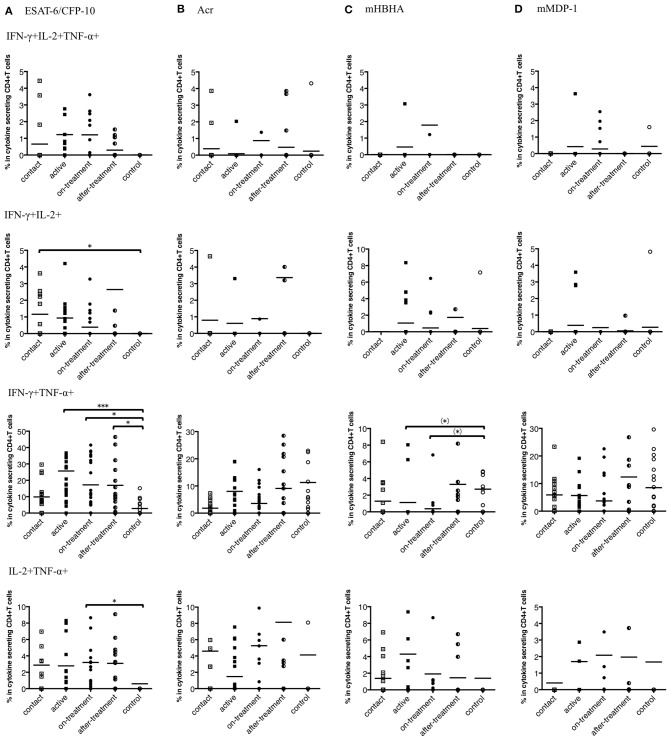
Percentages of polyfunctional CD4^+^ T cells in IFN-γ, IL-2, and TNF-α secreting CD4^+^ T cells to *M. tuberculosis*-associated antigens in *M. tuberculosis*-infected cases, grouped according to the disease stage as active TB cases (*n* = 24), on-treatment TB cases (*n* = 24), after-treatment TB cases (*n* = 19), and contact cases (*n* = 15). Responses of control cases (*n* = 18) are also shown. The differences between each set of samples were assessed using the Kruskal-Wallis test and *post hoc* Dunn's Comparison test (*P < 0.05, ***P < 0.001). (*) means the possible differences of preselected pairs (*P < 0.05). The long horizontal line represents the mean. **(A)** Against ESAT-6/CFP-10, three data points are outside the axis in IFN-γ, IL-2, and TNF-α positive CD4^+^ T cells, five data points are outside the axis in IFN-γ and IL-2 positive CD4^+^ T cells, five data points are outside the axis in IFN-γ and TNF-α positive CD4^+^ T cells, and seven data points are outside the axis in IL-2 and TNF-α positive CD4^+^ T cells. **(B)** Against Acr, two data points are outside the axis in IFN-γ, IL-2, and TNF-α positive CD4^+^ T cells, nine data points are outside the axis in IFN-γ and IL-2 positive CD4^+^ T cells, four data points are outside the axis in IFN-γ and TNF-α positive CD4^+^ T cells, and 18 data points are outside the axis in IL-2 and TNF-α positive CD4^+^ T cells. **(C)** Against methylated (m) HBHA, three data points are outside the axis in IFN-γ, IL-2, and TNF-α positive CD4^+^ T cells, one data point is outside the axis in IFN-γ and IL-2 positive CD4^+^ T cells, five data points are outside the axis in IFN-γ and TNF-α positive CD4^+^ T cells, and four data points are outside the axis in IL-2 and TNF-α positive CD4^+^ T cells. **(D)** Against mMDP-1, three data points are outside the axis in IFN-γ, IL-2, and TNF-α positive CD4^+^ T cells, one data point is outside the axis in IFN-γ and IL-2 positive CD4^+^ T cells, five data points are outside the axis in IFN-γ and TNF-α positive CD4^+^ T cells, and four data points are outside the axis in IL-2 and TNF-α positive CD4^+^ T cells.

Polyfunctional T cells that produce multiple cytokines provide more effective protective response against various infections and has been implicated in *M. tuberculosis* infection as well (17). We observed that the responses of IFN-γ and TNF-α positive CD4^+^ T cells against ESAT-6/CFP-10 were appreciably higher in after-onset TB cases (active, on-treatment, and after-treatment cases) (Figure 3A). Interestingly, this trend was not apparent when latency-associated *M. tuberculosis* antigens were applied (Figures 3B–D).

**Figure 4:**
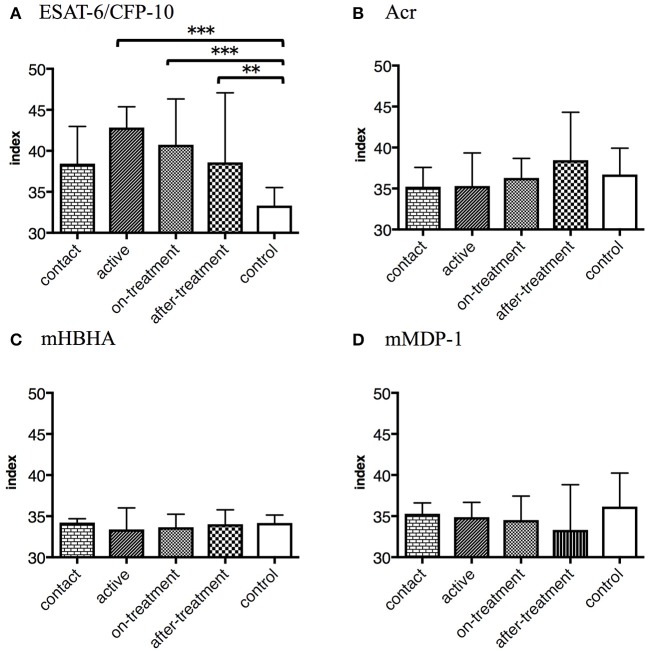
The values represent polyfunctional index of *M. tuberculosis*-associated antigens in *M. tuberculosis*-infected cases, grouped according to the disease stage as active TB cases (*n* = 24), on-treatment TB cases (*n* = 24), after-treatment TB cases (*n* = 19), and contact cases (*n* = 15). The values of control cases (*n* = 18) are also shown. Polyfunctional index of an object was calculated following an algorithm described previously (18). The differences between each set of samples were assessed using the Kruskal-Wallis test and *post hoc* Dunn's Comparison test (**P < 0.01, ***P < 0.001). The bar represents the median, and the vertical line and horizontal bar represents the interquartile range. **(A)** Against ESAT-6/CFP-10. **(B)** Against Acr. **(C)** Against methylated (m) HBHA. **(D)** Against mMDP-1.

Furthermore, we determined polyfunctional index, which reflects the degree and variation of polyfunctionality (18). We evaluated the responses of the polyfunctional CD4^+^ T cells to *M. tuberculosis*-associated antigens by employing this index. Against ESAT-6/CFP-10, index values were significantly higher in the after-onset (active, on-treatment, and after-treatment) TB cases than in the control cases (Figure 4A). However, no significant difference was observed against latency-associated *M. tuberculosis* antigens (Figures 4B–D).

### Interaction Between Cytokines in Response to *M. tuberculosis* Antigens

**Figure 5:**
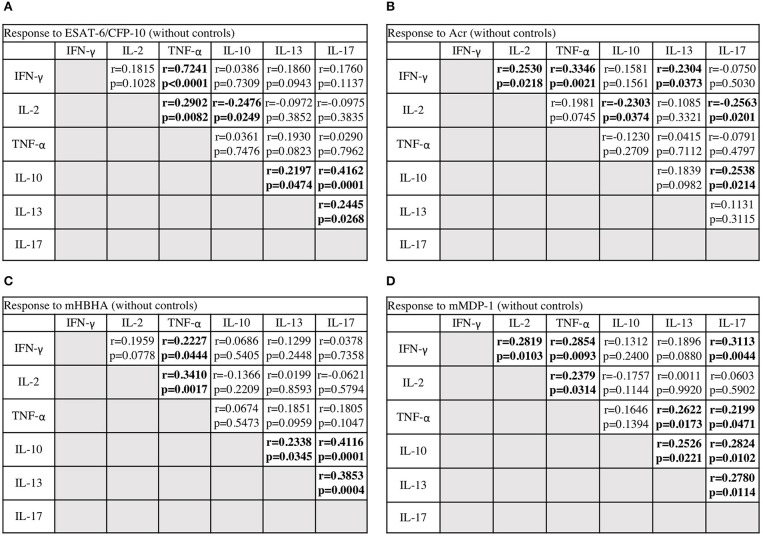
The correlations between cytokines in response to *M. tuberculosis* antigens among *M. tuberculosis*-infected cases. The correlations between each cytokine were assessed using Spearman correlation test. **(A)** Against ESAT-6/CFP-10. **(B)** Against Acr. **(C)** Against methylated (m) HBHA. **(D)** Against mMDP-1.

We systematically analyzed the correlation of diverse cytokine responses upon stimulation with different *M. tuberculosis* antigens among *M. tuberculosis*-infected cases (Figures 5A–D). As expected, strong correlation (*r* = 0.7241) was observed between Th1 cytokines against ESAT-6/CFP-10 and, to lesser extent but significant positive correlations (*r* = 0.2227–0.3410) were observed between Th1 cytokines against latency-associated antigens. Correlation between IFN-γ and IL-2 responses against ESAT-6/CFP-10 in *M. tuberculosis*-infected cases, grouped according to the clinical stage, showed that there were no correlations in active and on-treatment cases; however, there were moderate correlations in contact and after-treatment cases (data not shown, *r* = 0.568, *r* = 0.623, respectively). These results were consistent with those of the preceding study (19).

Conversely, there were significantly negative correlations between IL-10 and Th1 cytokine responses against ESAT-6/CFP-10 and Acr (*r* = −0.2476, *r* = −0.2303, respectively), and also between IL-17 and Th1 cytokine responses against Acr (*r* = −0.2563). In general, there were significantly positive correlations among IL10, IL-13, and IL-17 responses. We observed weak or no positive correlations between IL-13 and Th1 cytokine responses, and also between IL-17 and Th1 cytokine responses. These analyses demonstrated the independent nature of non-Th1 cytokine responses, particularly that of IL-10, from Th1 cytokine responses.

## Discussion

In the present study, we demonstrate that the level of Th1 cytokines, such as IFN-γ, IL-2, and TNF-α responses were dominant after TB onset (active, on-treatment, and after-treatment cases) regardless of the type of antigen. On the contrary, the notable levels of non-Th1 cytokines, such as IL-10, IL-13, and IL-17 responses were observed before TB onset (contact cases), and this trend was elucidated by *M. tuberculosis* latency-associated antigens, particularly apparent in IL-10 response against Acr as summarized in Table 2. This is the first study comprehensibly demonstrating the activation of non-Th1 cytokines by latency-associated antigens in comparison to ESAT-6/CFP-10 before TB onset and after TB treatment. Recently Pandey et al. reported the presence of IL-10 response against latency associated antigens (20), but they did not specify the type of cells by intracellular cytokine staining, neither did they show the responses against ESAT-6/CFP-10.

**Table 2:** Summary of cytokine responses.

	**Contact**	**Active**	**On Tx**	**After Tx**
**(A) Compared with control**				
ESAT-6/ CFP-10	IFN-γ^**^ IL-13^*^	IFN-γ^***^ IL-2^***^ TNF-α^***^	IFN-γ^***^ IL-2^***^ TNF-α^***^	IFN-γ^***^ IL-2^***^
Acr	IL-10^***^ IL-17^*^	IFN-γ^***^ IL-2^*^ TNF-α^***^ IL-10**	IFN-γ^***^ IL-2^***^ TNF-α^***^	IFN-γ^**^ IL-2^**^ TNF-α^**^
mHBHA	IFN-γ^*^ IL-17^**^	IL-2^**^ IL-17^***^	IFN-γ^***^ IL-2^***^ IL-17^**^	IFN-γ^**^ IL-2^***^ IL-17^**^
mMDP-1	IL-10(*)		IL-2^**^	IL-2^**^ IL-10(*)
**(B) Compared with contact**				
ESAT-6/ CFP-10		TNF-α^*^		
Acr		TNF-α^**^	TNF-α^**^ IL-10^**^	IL-10^**^
mHBHA			IL-2^*^	
mMDP-1			IL-2^**^	

The differences between each set of samples were assessed using the Kruskal-Wallis test and post hoc Dunn's Comparison test (*P < 0.05, **P < 0.01, ***P < 0.001). (*) means the possible differences of preselected pairs (*P < 0.05).

The most striking finding in the present study is that the level of CD4^+^ T cell responses such as IL-10 responses against Acr was the highest among contact cases. In fact, IL-10 responses against Acr were significantly higher compared to on-treatment and after-treatment TB cases. These correlations could not have been discovered in the previously published studies. The dynamics of IL-10 producing T cell response were independent of Th1 response (Figure 5) and these non-Th1 responses were more efficiently induced by latency-associated antigens. IL-10 is an anti-inflammatory cytokine, which suppresses the host immunity (21). The production of IL-10 is positively regulated by the cell signaling pathways, such as PI3K/AKT pathway (22). Some heat-shock proteins including Acr, and MDP-1, which are known to activate the PI3K/AKT pathway (23–25). *M. tuberculosis* produces both Acr and MDP-1 during the latent infection (8, 10). Especially, MDP-1 is expressed in the conditions mimicking inner-macrophage environment, such as hypoxia. Taken together our findings suggest that *M. tuberculosis* may uses tactic that exploits IL-10 producing T cells, such as regulatory T cells, to generate a favorable environment by impending the host immunity from attacking it and activating infected macrophages, allowing *M. tuberculosis* to survive. Because blood samples were tested in the present study, the T regulatory type1: Tr1 (26) might be a candidate of the IL-10 producing T cells.

Besides IL-10 responses, we also observed a significantly high IL-17 responses among contacts compared to that among controls, though the level was not higher than after-onset (active, on-treatment, and after-treatment) TB cases. IL-17 triggers the early inflammatory response via neutrophil recruitment (27). Acr and HBHA are shown to be distributed over the *M. tuberculosis* cell surface (9, 28) and HBHA acts as an adhesion molecule for the attachment of *M. tuberculosis* to alveolar epithelial cells. Thus, it is plausible that they encounter host immune system in the very early phase of *M. tuberculosis* invasion. Amir et al. reported Acr exposed dendritic cells produce IL-6 (29), which is a key cytokine for Th17 differentiation (30). Furthermore, IL-13 responses have also been observed among the contacts. IL-13 expression among TB cases were reported previously (31), but the implication of this phenomenon remains unknown. Recent studies indicated that the master regulators of CD4^+^ T cells–RORγt/Foxp3 or GATA3/Foxp3 are co-expressed (32–34). These CD4^+^ T cells are known to have high plasticity and can produce multiple cytokines. GATA3/ RORγt co-expressed CD4^+^ T cells are also indicated (35); thus, CD4^+^ T cells with high plasticity might produce both IL-13 and IL-17. Taken together, our findings of non-Th1 cytokines led us to hypothesize that mixed types of CD4^+^ T cells are induced by a different range of antigens during LTBI or prior to TB onset.

As previously described, the strongest IFN-γ response was observed in after-onset TB cases when stimulated with ESAT-6/CFP-10 but we reconfirmed our previous findings that not only IFN-γ but also IL-2 responses were dominant after TB onset when stimulated with multiple latency-associated antigens (36). TNF-α response was also dominant after TB onset when stimulated with latency-associated antigens. Intriguingly the frequency of IFN-γ responding to CD4^+^ T cells against ESAT-6/CFP-10 was one log higher than that against latency-associated antigens. This finding may reflect the fact that ESAT-6/CFP-10 is the most abundant during active TB owing to active replication of *M. tuberculosis*. ESAT-6/CFP-10 plays a pathogenic role by perforating the cell membrane of the alveolar epithelial cells or macrophages (37–39). The massive growth of *M. tuberculosis* within the macrophages leads to cell death. One type of cell death is apoptosis, which is also induced by ESAT-6/CFP-10 (40).

Polyfunctional T cells associated with TB have been studied earlier (17, 41–43), but no study showed polyfunctional T cells by latency-associated antigens. As IL-2 maintains the function of the memory T cells (44), we found apparent responses of the polyfunctional T cells with TB onset. We noted IFN-γ/IL-2 double positives in the contact cases, IFN-γ/TNF-α double positives in the active cases, and tri-functionals in the on-treatment cases. These results were similar to those of a previous study (17). Considering the fate of polyfunctional T cells (45), IFN-γ/TNF-α double positives are regarded as T cells that are optimized for effector functions; thus, Th1 cells with strong effector functions might be activated during early stages after TB onset. On the contrary, none of latency-associated antigens induced a demonstrable level of polyfunctional T cell responses with TB onset.

**Figure 6:**
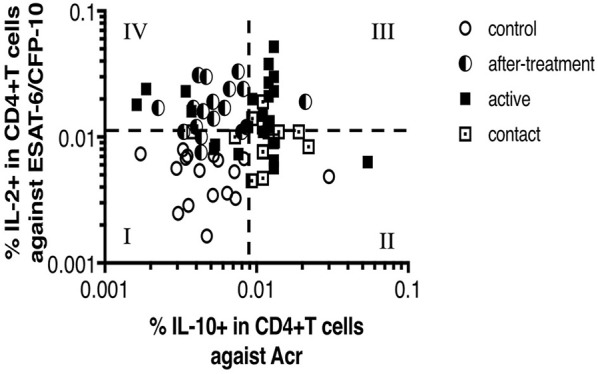
Scatter plot between IL-2 response against ESAT-6/CFP-10 and IL-10 response against Acr in *M. tuberculosis*-infected cases and control cases (*n* = 100, Two points are outside axis limits). This scatter plot was divided into four quadrates, and each quadrate was numbered from I to IV. Most of controls are in No. I quadrate. Contacts and actives are in No. II and No. III quadrates, in particular, actives are noticeable in No. III quadrate. After-treatments are noticeable in No. IV quadrate.

Finally, we hypothesized that the pattern of CD4^+^ T cell cytokine responses shift depending on the phase of *M. tuberculosis* infection owing to the abundancy of latency-associated *M. tuberculosis* antigen and active phase antigen stimulation. This also suggests the potential of latency antigens to be used for the early diagnosis of latent TB. We plotted a dot of our study individuals categorized into four groups of in two dimensions, IL-2 against ESAT-6/CFP-10 which represents Th1 type response to active phase TB antigens and IL-10 against Acr which represents non-Th1 response to latency-associated TB antigens in Figure 6. Controls cumulate in the left lower quadrant whereas active cases in the right upper quadrant. One of the striking findings is that after treatment cases clearly cumulate in the left upper quadrant. They were dissociated from active cases by IL-10 responses against Acr, while their IL-2 responses remained as high. This may reflect the living status of *M. tuberculosis* as the bacteria may exploit the stimulation of regulatory T cells as their survival strategy as discussed above. Although it is not apparent, contact cases tend to cumulate in the right lower quadrant. There could have been more contact cases in this quadrant, if they had not been selected by QFT positivity. On the other hand, there was one control in this quadrant. This individual might have been infected with *M. tuberculosis*. This raises a question whether the definition of contacts and controls determined by QFT alone is appropriate.

There are several limitations in the present study design. First, this present study was done in a cross-sectional manner. In order to reveal the shift in CD4^+^ T cell profile, we need to follow the patients in longitudinal manner. Second, we selected contacts from controls based on QFT results. As mentioned above, our contacts do not present the whole latent infections, which should include QFT negative cases. For further investigation, we need to design a study that takes account of the histories of close contacts or the endemic areas rather than QFT positively. Third, there was significant difference in the median age of each clinical case, especially between controls/contacts and other stages. We found no evidence of association of age by using linear regression analysis, however, possibility that aging has some influence on CD4^+^ T cells cannot be ignored completely. In Japan, *M. tuberculosis* non-infected cases or non-disease cases are limited to young generation. We carried out this study in the limited facilities of Japan and it was difficult to match the age in each clinical case. Lastly, we did not exclude TB patients with diabetes, which might have impaired immune responses. However, we prioritized recruiting a larger sample size because their diabetes was well-controlled and such effect was minimized. For our future study, recruiting more patients from other facilities would be required to match the ages.

In conclusion, non-Th1 cell responses to *M. tuberculosis* are preferentially induced by latency-associated antigens in LTBI. These findings raise a possibility of developing a new generation of cell-mediated immune assay to distinguish active TB infection from LTBI and/or cured TB, and bringing a new tool for improving management of *M. tuberculosis* infection.

## Materials and Methods

### Study Participants

*M. tuberculosis*-infected cases were recruited from four different hospitals in Japan, National Hospital Organization Higashi-saga Hospital, National Hospital Organization Nagasaki Kawatana Medical Center, Tagami Hospital (Nagasaki, Japan), and Nagasaki University Hospital.

Healthy controls with no history of TB were recruited at Nagasaki University.

All the blood samples included in this study were obtained with informed consent and with ethical approval from the Institute of Tropical Medicine Nagasaki University Joint Ethics Committee.

The *M. tuberculosis*-infected cases were divided into four clinical stages, active, on-treatment, after-treatment, and contact. The *M. tuberculosis*-infected cases without contact cases were clinically diagnosed and confirmed by using smear PCR, culture, or course of treatment. Active cases were at the point from the diagnosis to within 10 days treatment. On-treatment cases were at the point after 10 days treatment. The QFT test (QuantiFERON®TB-GOLD In-Tube; QIAGEN, Hilden, Germany) positive cases without any symptoms or history of TB were regarded as contact cases. The QFT test intermediate cases that had a history of contact with TB patients were also regarded as contact cases. The QFT test negative cases without any symptoms were considered as healthy controls.

### Reagents

ESAT-6, CFP-10, Acr, HBHA, and MDP-1 were recombinant protein products from *E. coli*. The synthesis of these proteins was carried out as described previously (36). Chemical methylation in HBHA and MDP-1 was also performed because we ascertained the necessity of methylation previously (36).

The following fluorescently-labeled monoclonal antibodies were used in this study: anti-CD3-APC-Cy7 (HIT3a), anti-IFN-γ-PE-Cy7 (4S.B3), anti-IL-10-PE (JES3-9D7), anti-IL-17-Alexa Fluor 700 (BL168), anti-TNF-α-PerCP-Cy5.5 (MAb11) (Biolegend, San Diego, CA, USA), Anti-CD4-Pacific Blue (OKT4), anti-IL-2-APC (MQ1-17H12), and anti-IL-13- FITC (PVM13-1) (eBioscience, San Diego, CA, USA). Cell viability was assessed using a LIVE/DEAD kit (Invitrogen, Carlsbad, CA, USA). FcR blocking reagent was purchased from MBL (Nagoya, Japan). CD28/CD49d co-stimulator was purchased from BD Bioscience (San Jose, CA, USA). Brefeldin-A (BFA) and Monensin sodium salt were purchased from Sigma-Aldrich (St. Louis, MO, USA) and Wako Junyaku Co. Ltd (Tokyo, Japan), respectively.

### *In vitro* Culture

Peripheral blood mononuclear cells (PBMCs) isolation and culture were performed as described previously (36). The PBMCs were isolated within 6 h after taking heparinized blood samples. Antigen concentrations were as follows: ESAT-6 and CFP-10 (0.75 μg/ml each), Acr (0.8 μg/ml), mHBHA (1.7 μg/ml), and mMDP-1 (6 μg/ml). Cultures with no antigen (medium only) were also included. All antigen stimulations were performed in the presence of CD28/CD49d co-stimulator (0.5 μg/ml) and golgi-blocker: BFA (1 μg/ml) and Monensin (0.5 μM). Cell incubation was overnight (14–16 h) at 37°C in a 5% CO_2_ incubator.

### Flow Cytometry

Cell surface staining, permeabilizing, and intracellular cytokine staining were performed as described previously (36). At surface staining stage, 30% goat serum containing anti-CD3, anti-CD4, FcR blocking reagent, and LIVE/DEAD reagent were added. At intracellular cytokine staining stage, anti-cytokine monoclonal antibody cocktail containing IFN-γ, IL-2, IL-10, IL-13, IL-17, TNF-α, and FcR blocking reagent were added. Cells were acquired using Gallios (Beckman Coulter, Brea, CA, USA). Flow cytometry data were analyzed using FlowJo software, version 8.8.7 (TreeStar, San Carlos, CA, USA). Gating strategy for flow cytometric analysis are shown in Supplementary Figure 1.

### Polyfunctional Index

Polyfunctional index of an object was calculated following an algorithm described previously (18). Then, group medians of these indexes were compared.

### Statistical Analysis

Group medians and distributions were analyzed using Kruskal-Wallis test with *post-hoc* Dunn's Comparison test. Associations were adjusted by using Linear regression analysis. Correlations were analyzed using Spearman correlation test. Analysis were performed with GraphPad Prism software, version 5 and 7 (San Diego, CA, USA) and STATA software, version 13 (College station, TX, USA). The threshold for significance was *P* < 0.05.

## Data Availability Statement

The datasets generated for this study are available on request to the corresponding author.

## Ethics Statement

The studies involving human participants were reviewed and approved by the Institute of Tropical Medicine Nagasaki University Joint Ethics Committee. The patients/participants provided their written informed consent to participate in this study.

## Author Contributions

YY designed the study, mainly performed laboratory investigation, performed the statistical analysis, and prepared the manuscript. MO-O, YO, YT-Y, and SM helped designing the study. TO, KK, and KT helped laboratory investigation. IY and TE helped and verified the statistical analysis. TT and KA edited the manuscript. All authors contributed to manuscript revision, read and approved the submitted version.

### Conflict of Interest

The authors declare that the research was conducted in the absence of any commercial or financial relationships that could be construed as a potential conflict of interest.

## Abbreviations

*M. tuberculosis*: *Mycobacterium tuberculosis*
TB: Tuberculosis
LTBI: Latent *M. tuberculosis* infections
Th: T-helper
IGRAs: IFN-γ release assays
QFT: QuantiFERON TB
Acr: α-crystallin
HBHA: Heparin-binding hemagglutinin
MDP-1: Mycobacterial DNA-binding protein 1
m: methylated.
