# Supplementary material for: CD4+ T Responses Other Than Th1 Type Are Preferentially Induced by Latency-Associated Antigens in the State of Latent Mycobacterium tuberculosis Infection
Source: Front Immunol. 2019 Nov 29;10:2807. doi: 10.3389/fimmu.2019.02807 (PMC6897369; doi:10.3389/fimmu.2019.02807)
Supplement: Supplementary file 1 [file Data_Sheet_1.docx]

Supplementary Material

# Supplementary Figures

**Supplementary Figure 1.** (A) Gating strategy for flow cytometric analysis of CD4^+^ T cells. PBMCs were prepared from the blood samples. Cell surface was stained with fluorescently labeled monoclonal antibodies against CD3 (APC-Cy7), and CD4 (Pacific Blue). Intracellular staining was performed with fluorescently labeled monoclonal antibodies against IFN-γ (PE-Cy7), IL-2 (APC), TNF-α (PerCP-Cy5.5), IL-17 (Alexa Fluor 700), IL-10 (PE), and IL-13 (FITC). Data were collected with Gallios and analyzed with FlowJo software. Lymphocytes were identified by scatter properties (forward scatter × side scatter plot), and then, dead cells were excluded. The surface CD3^+^ cell represents T lymphocyte. From the lymphocytes, the CD4^+^ cells were chosen. (B) Representative flow cytometry results showing IFN-γ, IL-2, TNF-α, IL-17, IL-10, and IL-13 responses of CD3^+^CD4^+^ cells against Concanavalin A stimulation. (C) Representative flow cytometry results showing IFN-γ responses of CD3^+^CD4^+^ to various *M. tuberculosis-*associated antigens.
